# Perspective on Constructing Cellulose-Hydrogel-Based Gut-Like Bioreactors for Growth and Delivery of Multiple-Strain Probiotic Bacteria

**DOI:** 10.1021/acs.jafc.1c00468

**Published:** 2021-04-23

**Authors:** Srinivas Mettu, Zubeen Hathi, Sandya Athukoralalage, Anshu Priya, Tsz Nok Lam, Khai Lun Ong, Namita Roy Choudhury, Naba Kumar Dutta, Rodrigo Curvello, Gil Garnier, Carol Sze Ki Lin

**Affiliations:** ‡ School of Energy and Environment, City University of Hong Kong, Tat Chee Avenue, Kowloon, Hong Kong; § Chemical and Environmental Engineering, School of Engineering, RMIT University, Melbourne, Victoria 3000, Australia; ∥ Bioresource Processing Institute of Australia (BioPRIA), Department of Chemical Engineering, Monash University, Clayton Victoria 3800, Australia

**Keywords:** biotherapeutics, cellulose hydrogels, encapsulation, gut microbiota, hydrogel bioreactors, probiotic bacteria

## Abstract

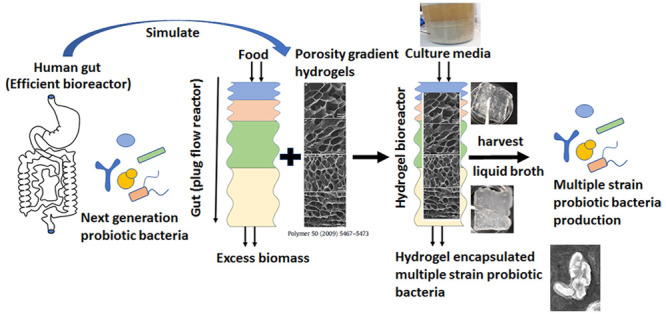

The current perspective presents an outlook on developing gut-like bioreactors with immobilized probiotic bacteria using cellulose hydrogels. The innovative concept of using hydrogels to simulate the human gut environment by generating and maintaining pH and oxygen gradients in the gut-like bioreactors is discussed. Fundamentally, this approach presents novel methods of production as well as delivery of multiple strains of probiotics using bioreactors. The relevant existing synthesis methods of cellulose hydrogels are discussed for producing porous hydrogels. Harvesting methods of multiple strains are discussed in the context of encapsulation of probiotic bacteria immobilized on cellulose hydrogels. Furthermore, we also discuss recent advances in using cellulose hydrogels for encapsulation of probiotic bacteria. This perspective also highlights the mechanism of probiotic protection by cellulose hydrogels. Such novel gut-like hydrogel bioreactors will have the potential to simulate the human gut ecosystem in the laboratory and stimulate new research on gut microbiota.

## Introduction

The microbial ecosystem present in the human gut significantly affects the health of the human host.^1^ Some strains from this microbial community termed as “probiotics” are consumed live to promote health benefits and keep the gut mucosal layer free from invading pathogens, thereby boosting the immune system. This microbial community performs multiple functions, including nutrient metabolism, stimulation of the immune system, regulation of metabolic functions, and even defense of the host from pathogens. The universally accepted narrative of probiotics given by the Food and Agriculture Organization (FAO)^2^ of the United Nations states that “probiotics are live microorganisms, which when administered in adequate amounts confer a health benefit on the host”. Earlier research has demonstrated the efficiency of probiotics in curing symptoms related to several bowel disorders, gastrointestinal (GI) inflammations, and diarrhea.^3^ Microbial count [colony forming unit (CFU)] and viability are among the essential conditions for the microbes to have the desired effect on human health.^2,3^ Viable microbes that produce useful metabolites to the host are considered as probiotics, which are known to exert positive ramifications on host health by offering protective barriers, exclusion of pathogenic microbes in the GI tract, and augmentation of the baseline immune response.^4^

The application of microbes in food and beverages has a long-celebrated history with society initially discovering the benefits of consuming fermented foods. Naturally or commercially produced probiotic bacteria consumed by the majority of the population in the form of simple yogurt are directly associated with the early ferments. The apparent health advantages of probiotics have led toward their inclusion in a large variety of food and beverages, including cereals, cheese, ice cream, and milk shakes.^5^ However, concerns related to their functional viability and delivery to the intended location of the human gut still persist.^6^ Evidently, for any probiotic to have beneficial effects, the bacterial suspension must pass and survive through the digestive tract and reach the colon in substantial amounts.^7,8^ According to a World Health Organization (WHO) report, the minimum number of viable probiotic bacteria in any food supplement to be retailed with health claims is 10^6^–10^7^ CFU.^9^ Moreover, there are other variables, including pH, oxygen, and temperature, that apparently affect the viability of the bacterial suspension administered as a probiotic.^10−12^

Numerous studies have been carried out to investigate the role of different encapsulation vehicles in protecting the viability of cultures throughout processing, delivery, storage, and GI transit.^13−16^ Carrier systems based on proteins, biopolymers, and lipids are considered as excellent encapsulation vehicles.^17^ Protein-based encapsulated probiotic formulations are composed of several animal- and plant-derived proteins, such as gluten, zein, gelatin, and milk proteins. Probiotic encapsulation in plant-derived vegan proteins is being considered as an excellent alternative to animal-derived proteins.^13,18^ Lipids, fats, oils, waxes, and resins are another category of biomaterials considered as a favorable matrix for encapsulation of probiotics.^19,20^

However, encapsulation systems based on conventional biopolymers, such as alginate, have some limitations in protecting the probiotics from gastric fluids.^21−25^ Cellulose-based hydrogels on their own or in combination with other biopolymers have recently shown great promise to overcome the limitations of conventional biopolymer-based encapsulation systems.^21,26−33^

**Figure 1:**
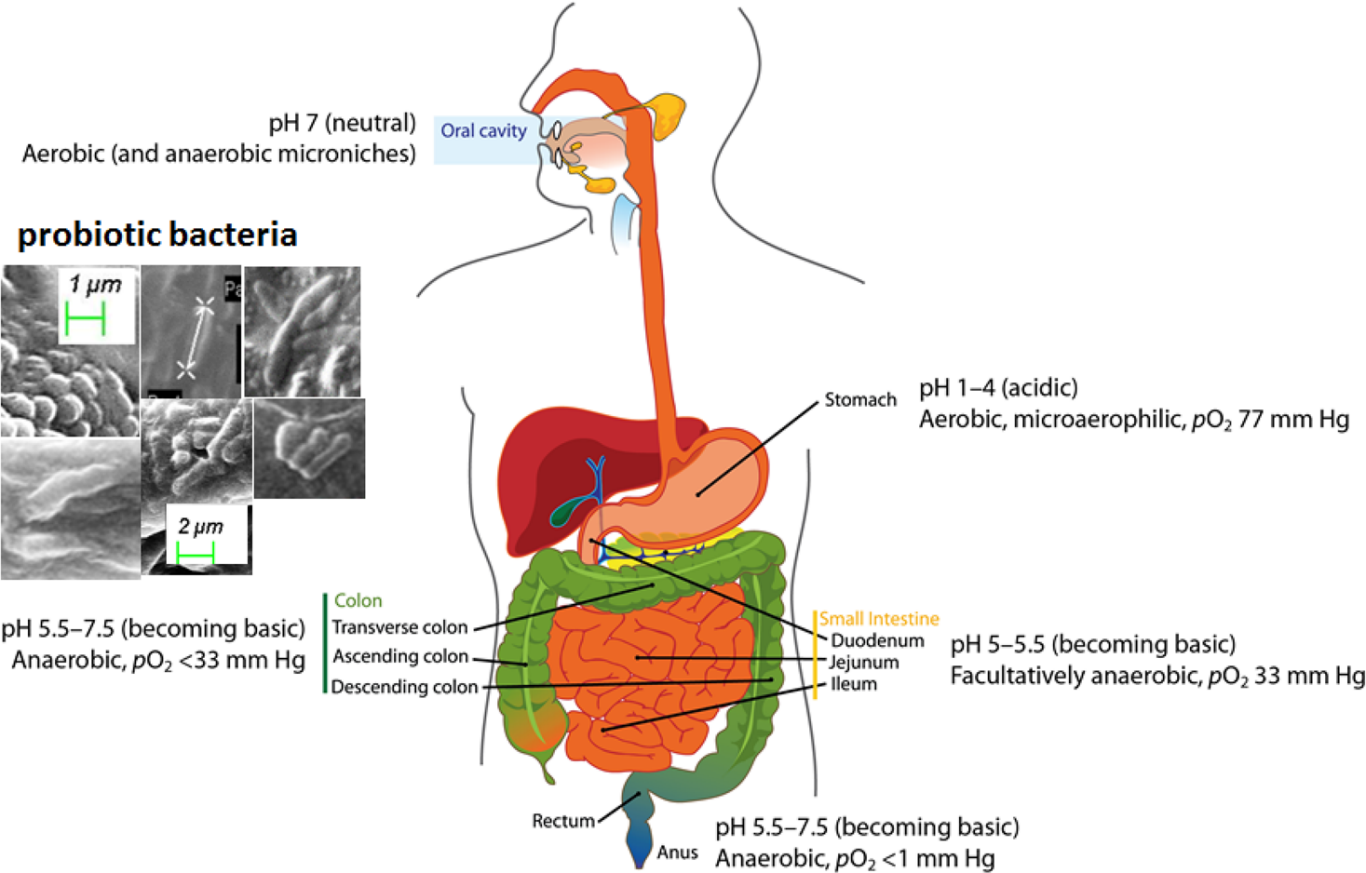
pH and oxygen level distribution in the human digestive tract along with the type of gut bacteria that grow in various regions. The image was adapted with permission from ref (48) and modified by adding the scanning electron microscopy (SEM) pictures of a few anaerobic probiotic bacterial cultures in our laboratory. The strains are *Ruminococcus bromii* (RB), *Terrisporobacter glycolicus* (TG), *Roseburia intestinalis* (RI), *Faecalibacterium prausnitzii* (FP), *Bacteroides salyersiae* (BS), and *Collinsella aerofaciens* (CA). The oxygen levels are obtained from refs (48 and 49).

While most of the existing literature focuses on delivery of single and multiple common probiotic strains belonging to *Lactobacillus* and *Bifidobacterium* genera, for health benefits, there is emerging evidence that multiple, difficult to culture, strictly anaerobic, novel probiotic strains from other phyla, such as Firmicutes, Bacteroidetes, and Actinobacteria, may be needed (Figure 1).^34^ Recent literature suggests that, in children, gut microflora gets destroyed when they are malnourished.^35−40^ To restore it, they need to be administered with multiple strains of aerobic and anaerobic microbes that colonize the gut regions from the small intestine to rectum (Figure 1). However, it is expensive to produce multiple stains of microbes in individual bioreactors.^34^ Hence, there is an urgent need for developing novel bioreactors that can produce multiple strains. Growth of gut microbial consortia is possible only when the bioreactor can simulate the human gut environment in it (pH and oxygen gradients as shown in Figure 1). Hydrogels derived from cellulose nanofibers are suitable to address this challenge because they can be chemically altered to control porosity and water retention capacity.^41−47^ These properties can be used to create and maintain gradients of oxygen and pH across the hydrogel material. Such a possibility makes cellulose hydrogels an ideal contender for constructing gradient bioreactors. The current novel hypothesis is that cellulose-based hydrogels with gradient structure and controlled porosity can be used to control pH and oxygen level, thereby simulating the human gut environment in bioreactors. Multiple strains of human gut microbes can be produced in these bioreactors, where the bacteria-immobilized hydrogels can be used as a probiotic delivery vehicle into the human colon.

The current perspective presents novel concepts on developing gut-like bioreactors with immobilized probiotic bacteria using cellulose hydrogels. The gut-like bioreactor can be used for not only production but also delivery of multiple-strain probiotics to the human colon using immobilized growth of bacteria on hydrogels. We describe some novel trends and promising results in probiotic encapsulation using cellulose-based hydrogels for controlled delivery to the colon.

## Prospects of Using Cellulose Hydrogels for Production of Multiple-Strain Probiotics

Recent literature suggested that severely malnourished children do not recover to full health even after they are fed well later in their lives.^35−40^ Their brains did not develop to their full potential, and the children were susceptible to diseases in later stages of their lives.^37−39^ It has been clearly identified that the optimal functioning of the gut requires a specific microbial community because its coupling with suitable changes in the dietary habits of the host generates the required energy for the host to deliver health benefits. Restoration of the gut microbiome has the potential to offer these children healthier lives. However, probiotic bacteria products available in the market contain only a few easy-to-grow strains from the *Lactobacillus* and *Bifidobacterium* genera, which constitute only a small fraction of the gut microbiome. To restore the overall loss in microbial diversity caused as a result of either gut dysbiosis or malnutrition, it is essential to reconstitute the gut microflora by administering multiple strains of aerobic and anaerobic bacteria that belong to other phyla of human gut microbes.

The general practice of growing multiple strains of bacteria nowadays is to culture them in separate bioreactors using liquid media for growth because they require different nutrients, growth media, and oxygen levels (Figure 1). However, growing each strain in a different bioreactor makes the production process complicated and costly to maintain. For example, multiple-strain probiotics available in Australian supermarkets cost around ∼AU$1 per dose. Such a high cost is not affordable for malnourished children in underdeveloped and developing countries. Based on recent scientific evidence from studies on malnourished children^37−39^ and the need to reduce the cost of production, there was a call from the Bill and Melinda Gates Foundation as a part of the Grand Challenges Exploration (GCE) initiative in Round 22 titled “New Approaches for Manufacturing Gut Microbial Biotherapeutics”,^34^ which highlights the importance and significance of this area of topical research. To tackle this issue and to reduce the cost of production, there is a need to construct hydrogel-based gut-like bioreactors that can simulate the pH and oxygen (pO_2_) gradients found across the human gut,^49−51^ enabling the growth of multiple strains of aerobic and anaerobic bacterial species simultaneously (Figure 1).

In this perspective, we present the ideas and rationale behind the proposed reactor using cellulose hydrogels and how it is beneficial not only to produce multiple strains of probiotic bacteria in a single bioreactor but also for encapsulation and controlled delivery. With this perspective, we aim to widely disseminate the novel ideas among the researchers working on cellulose hydrogels to draw their attention to accelerate this novel research field and direction.

## Human Gut as a Compartmentalized Bioreactor

**Figure 2:**
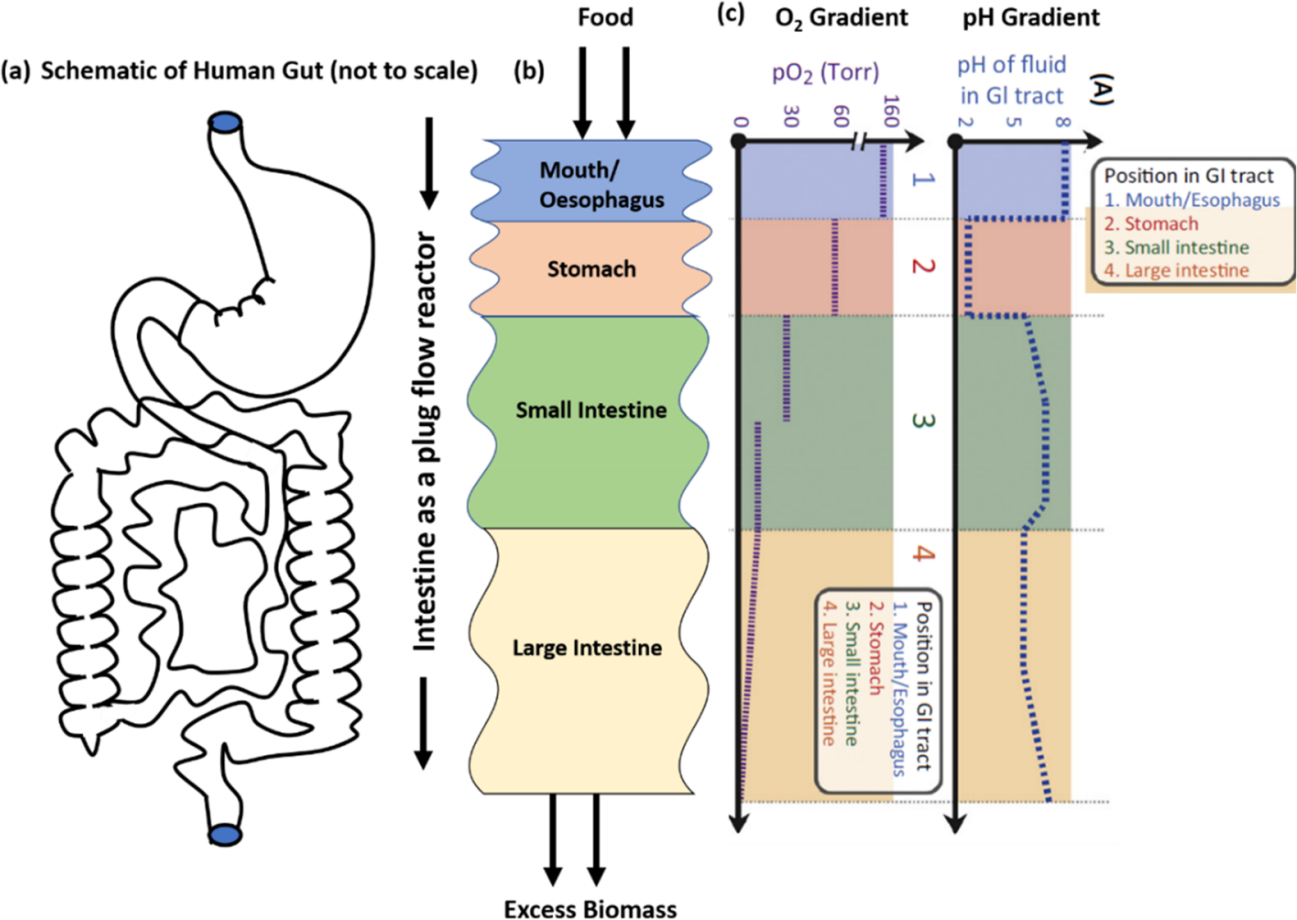
Human gut as a plug flow reactor having gradients in pH and pO_2_ across the intestinal tract.^50^ The figure depicts the change in pO_2_ and pH levels from the stomach to the distal gut.^51^ This figure was redrawn and adapted with permission from ref (51). Copyright 2019 Nature Publishing Group.

The human GI tract is responsible for food digestion, nutrient absorption, secretion, and motility of undigested parts for excretion.^52^ Apart from digestive functions, the enteric nervous system that spans the whole gut confers an indirect effect on mental and physical well-being through the gut–brain axis. The human gut is also home to more than 100 trillion commensal microorganisms.^53^ The composition of these gut microbes significantly affects our health. As shown in Figures 1 and 2, various microbes colonize in different parts of the gut, from the stomach to the rectum. The colonization region of gut microbes depends upon their oxygen sensitivity. The microbial composition changes from mostly aerobic bacteria in the mouth (pH ∼ 7 and pO_2_ ∼ atmospheric) to microaerophilic bacteria in the stomach (acidic pH ∼ 1–4 and pO_2_ ∼ 77 mmHg). In the small intestine, the pH becomes increasingly basic (pH ∼ 5–5.5) and the oxygen level drops even more (pO_2_ ∼ 33 mmHg); therefore, facultative anaerobic bacteria grow in this region. Further down the colon and rectum, pH increases further, reaching values of greater than 7, and the oxygen level (i.e., pO_2_) drops below 33 and 1 mmHg in the colon and near the rectum, respectively. The approximate pH and pO_2_ values are shown in Figures 1 and 2.^50,51^

Because the human gut microbiome contains multiple strains of aerobic, microaerophilic, and anaerobic bacteria, it becomes quite essential to have all of these different types of strains while developing probiotics for biotherapeutic interventions. However, because these various strains need very different nutrient supply, pH, and oxygen levels, they cannot be grown in a single bioreactor. Multiple strains are generally grown in separate bioreactors, extracted, freeze-dried, and then mixed later in dry form. This process makes multi-strain probiotic-based health interventions expensive. However, from a chemical engineering point of view, the gut can be treated as a compartmentalized plug flow reactor (Figure 2).^52^ Such an idealization of regions makes it easy to design a bioreactor with various compartments interconnected, as in the human gut, that can have crosstalk between them and to simulate realistic GI conditions in the reactor. The design of the reactor can be used to control pH and pO_2_ gradients in the reactor to match human gut conditions. Once the control of the physical gradients is achieved, the bioreactor can be seeded with various aerobic and anaerobic gut microbes with biotherapeutic potential. The seeding can be performed with individual strains in different parts of the reactor with a favorable growth environment or multiple strains can be seeded together as a mixture. The specific microbes will grow in their comfort zones of a favorable environment, including pH and pO_2_ values. Bioreactors that can accommodate such a huge variation in the microbial environment are difficult to design. However, it can potentially be achieved using the optimized design of immobilization materials with varying degrees of spatial structuring and gradients.

Cellulose hydrogels are promising immobilizing materials with potential to achieve this goal. They are manufactured from plant- or bacterial-based cellulose nanofibrils, making them biodegradable, renewable, hydrophilic, and biocompatible.^54^ Plant-based cellulose biomass is abundantly available all over the world. Several inorganic immobilization materials are not suitable for this application because the resultant food products are intended for human consumption. Hence, it is essential to use food-grade biopolymers that are derived from plant sources.

The cellulose-based hydrogels can be chemically altered using functional groups or by incorporating with active biomolecules. The cellulose hydrogel structure formation is due to the natural association of nanofibers in combination with electrostatic stabilization that can be used to control porosity. The porosity can be varied by concentration and mixing ratios of various forms of cellulose, such as cellulose nanofibers (CNFs) and cellulose nanocrystals (CNCs). The stability of these hydrogels can be further improved by chemical cross-linking that leads to flexibility and tenacity to the structure.^55^ These characteristics of cellulose hydrogels make it an ideal contender in constructing a gut-like bioreactor, because porosity and cross-linking density of these hydrogels can be altered to achieve spatial control over pH, oxygen, and nutrient levels. We also argue that the hydrogels used in the construction of the gut-like bioreactor must be food-grade, so that the hydrogels can be used for encapsulation. To our knowledge, this is the first report that discusses and develops novel concepts for the growth of both aerobic and anaerobic microbial strains of the human gut in a single bioreactor system.

## Use of Cellulose-Based Hydrogels for Probiotic Production

Plant-based cellulose-derived products, such as CNFs and CNCs are major classes of this material, which are generally modified or combined with other materials to impart desired chemical properties. CNFs generally have a diameter of about 5–50 nm, and the fiber length can be more than 1 μm.^56^ The long aspect ratio (>100) imparts flexibility to the fibers, so that it can form an interconnected network when dissolved in water, resulting in hydrogels of reasonable strength at low concentrations (<1 wt %) without external chemical cross-linking. On the other hand, CNCs are rigid short crystals, which typically have low aspect ratios (<30), resulting in rigid morphology. As a result of the rigid crystalline nature, CNCs require high concentrations to form hydrogels of reasonable strength. However, as a result of the completely different characteristics of CNFs and CNCs, they can be mixed in various concentration ratios to obtain hydrogel of controlled physicochemical characteristics. For example, if a highly porous hydrogel network is needed, CNFs can be used. While if rigid and low porosity hydrogel is needed, CNCs can be used. Therefore, regulation of the concentration and ratio of CNFs and CNCs in a gel system can be used to tune the porosity and rigidity of hydrogels. Surface chemistry modifications and introducing cross-linking networks are the possible methods to achieve mechanically stable CNC hydrogels because CNCs alone cannot form stable hydrogels as a result of the small aspect ratio and rigid structure.^57^

Both CNCs and CNFs can be physically and chemically entrapped into polymeric hydrogel matrices to improve their mechanical properties. Rigidity of CNCs allows them to efficiently act as fillers in polymeric hydrogel composites. More flexible and soft hydrogels could be obtained by incorporating CNFs. However, loading ability of CNFs is lower than that of CNCs as a result of the tendency for entanglement. Different methods, such as homogenization,^58^ free radical polymerization,^59^ solution casting,^60^ freezing/thawing cycle,^61^ three-dimensional (3D) printing,^62^ and ultraviolet (UV)/ion-mediated cross-linking,^63^ have been extensively studied to incorporate CNCs and CNFs into polymeric hydrogel networks.

## Existing Literature Data on Synthesizing Cellulose-Based Hydrogels with Controlled Porosity and Water Retention Capacity

The important characteristics of cellulose hydrogels required to maintain pH and O_2_ gradients for developing a gut-like bioreactor are controlled porosity and water absorption capacity. Another essential characteristic is that the hydrogels must be food-grade. Below, we discuss a few important studies from recent literature that have attempted to synthesize cellulose-based hydrogels with controlled porosity and water absorption capacities, albeit using harsh chemicals, which make them unsuitable to be used for human consumption or in food-grade products. We address this issue of chemical-free synthesis of food-grade cellulosic materials later. Although the studies described below used non-food-grade chemicals in the synthesis, they still provide the necessary background information needed to support the hypothesis that cellulose hydrogels, in theory, can be used to control pH and O_2_ gradients needed to construct a novel gut-like hydrogel bioreactor.

**Figure 3:**
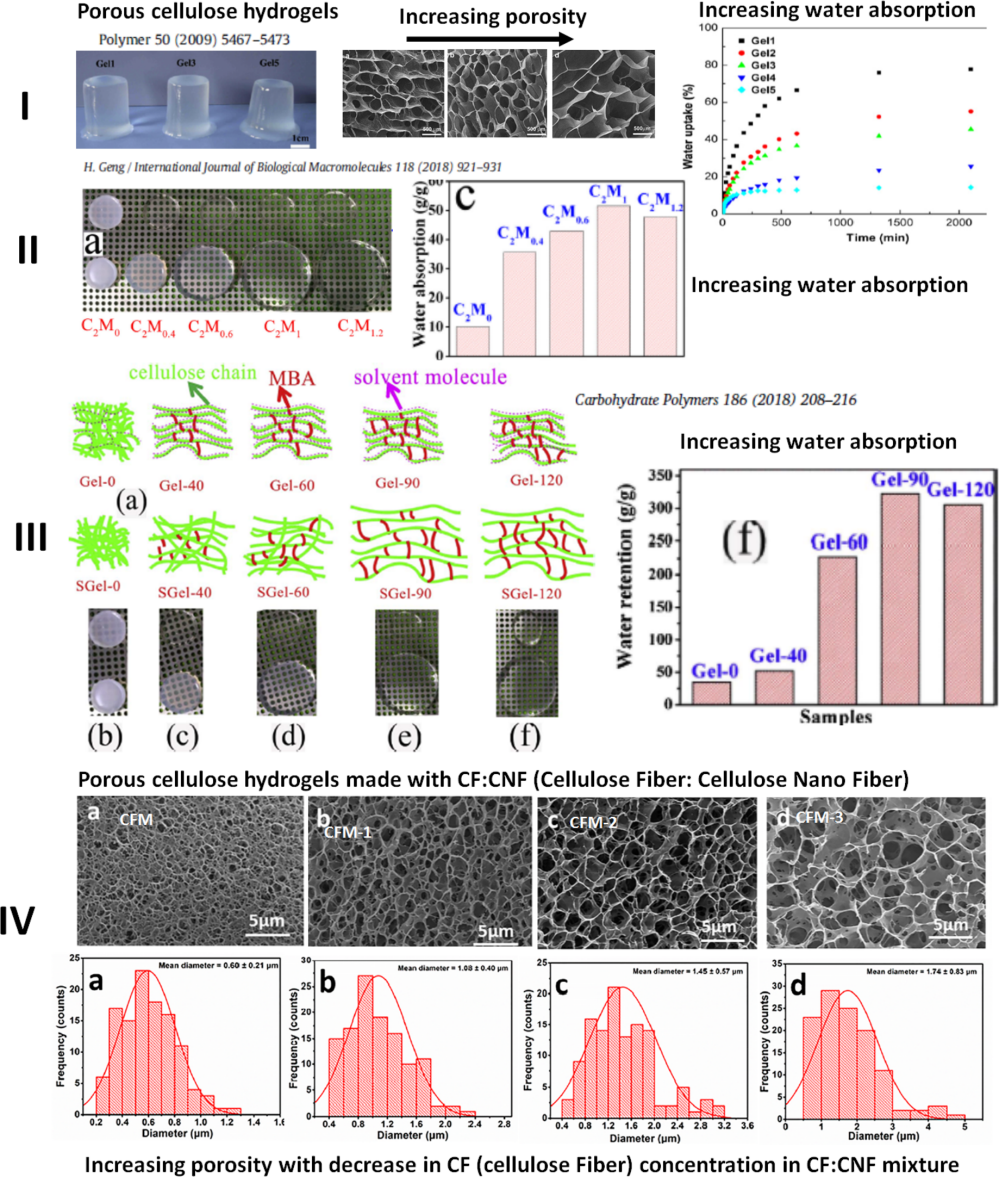
Examples of controlled porosity hydrogels synthesized by various researchers.^26,41−47^ Controlled porosity cellulose-based hydrogels have already been shown to have various water absorption capacities.

Many researchers have attempted to combine cellulosic materials with other biodegradable polymers and biopolymers, such as sodium alginate and other proteins.^41−47,64,65^ We present the data obtained in studies by Chang et al.,^42^ Geng,^46,47^ and Luan et al.^26^ in Figure 3 to explain how various approaches have been used to tune the porosity and water absorption. Although this list is not exhaustive, it provides a glimpse of what has been tried thus far. Chang et al.^42^ have used epichlorohydrin (ECH) to cross-link a blend of cellulose and alginate solution to synthesize hydrogels with tunable macroporosity and water absorption. It was found that the gel became stiff, porosity decreased, and water absorption increased with an increasing concentration of cellulose in the blend (row I of Figure 3). Geng^46,47^ has used *N*,*N*-methylene bis(acrylamide) (MBA) as a cross-linking agent to prepare micro- and macroporous hydrogel by increasing the concentration of MBA. It was found that with increasing MBA concentration, the gels became stiffer, more transparent, and porous and water absorption increased (rows II and III of Figure 3). Geng^46^ has presented a schematic model of how the solvent (NaOH/urea) and cross-linker MBA result in the formation of an entangled cellulose network as a result of hydrogen bonding in the absence of a cross-linker, resulting in low porosity and transparency, whereas the inclusion of MBA in between the cellulose molecules increases transparency, stiffness, porosity, and water absorption with increased MBA concentrations. The reader is referred to studies by Geng^46,47^ for further details. Luan et al.^26^ have used a combination of native cellulose fiber (CF) and CNF to prepare cellulose macrogel particles by extrusion into hydrochloric acid (HCl), as shown in row IV of Figure 3. It was found that with an increase in the CNF concentration, the average porosity increased from 0.6 to 2.1 μm. As shown in SEM images in Figure 3, the porosity of hydrogels can be tuned by either changing the nature of cross-linking, cross-link density, or concentrations of cellulose/alginate. However, the main limitation of using these hydrogels for growth of probiotic bacteria comes from the fact that toxic chemical cross-linkers, such as ECH and MBA, and catalyst, such as 2,2,6,6-tetramethylpiperidine-1-oxyl radical (TEMPO), were used. Hence, there is a critical need for developing non-toxic cross-linking methods for preparing such hydrogels. A recent study by Varanasi et al.^66^ that synthesized CNFs from carrot pomace using chemical-free methods offers a hope for preparing food-grade cellulose hydrogels from either agricultural or food waste.

Apart from chemical cross-linking methods to control porosity, there exists an array of physical methods that can be used to regulate porosity.^56,67^ Most of these physical methods are used in preparation of cellulose-based foams (aerogels and cryogels), but they can be used for hydrogels as well. For example, during the preparation of hydrogels, a high shear force can be used to trap air/gas bubbles of controlled size inside the hydrogel solution, which can act as a template for creating pores.^68^ Similarly, oil-in-hydrogel or hydrogel-in-oil emulsion^69^ can be prepared with a specified emulsion drop size, which can be later removed by solvent extraction, leaving a specific pore structure. An ice crystal templating method can also be used, where controlled freezing of water can create pores.^70^ The existing experimental results of porosity tuning of cellulose demonstrate the potential of using them to simulate an intestinal environment.

## Recent Advances in Generating Immobilized pH Gradients Using Polyacrylamide Hydrogels

**Figure 4:**
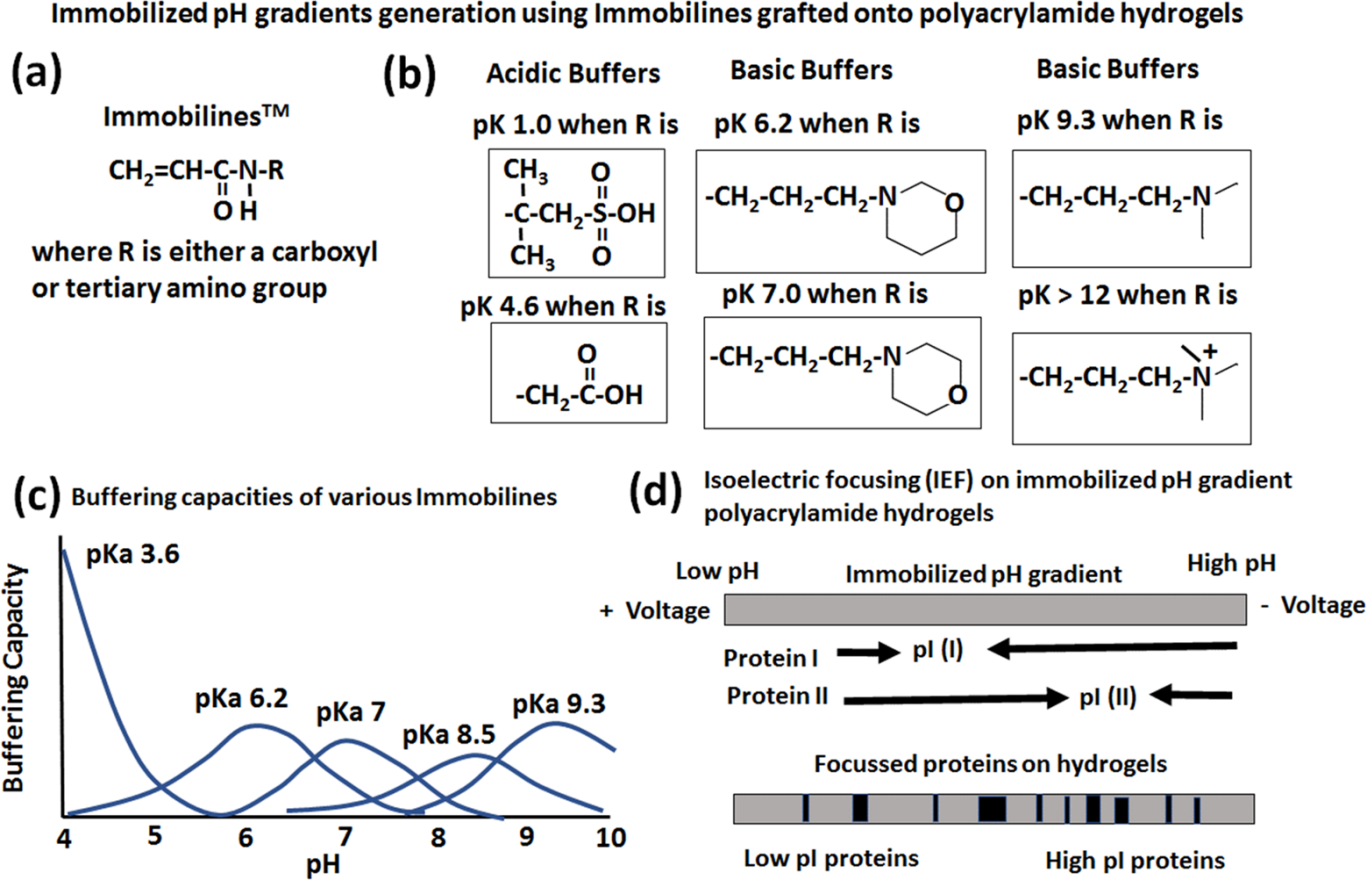
Existing approaches in the literature^71−74^ on creating immobilized pH gradients using immobilines covalently grafted onto polyacrylamide gels and their use in isoelectric focusing for protein separation. This figure is adapted and redrawn with permission from refs (71−74).

Generation of pH gradients inside the proposed hydrogel-based bioreactor is essential for simulating the human gut environment and multi-strain probiotic production. Immobilized pH gradients generated using immobilines covalently grafted onto polyacrylamide gels with their use in isoelectric focusing for protein separation is a well-established technology, which is reviewed in detail by many authors.^71−74^ The details of the immobiline structure along with some examples of acidic and basic acrylamido buffers and their use to generate immobilized pH gradients for isoelectric focusing of proteins are shown in detail in Figure 4. The reader is referred to excellent reviews for further details.^71−74^ Our approach of using only cellulose hydrogels along with buffers is presented in the next section.

## Proposed Approach on Generating Immobilized pH and Oxygen Gradients in Using Cellulose-Based Hydrogels

**Figure 5:**
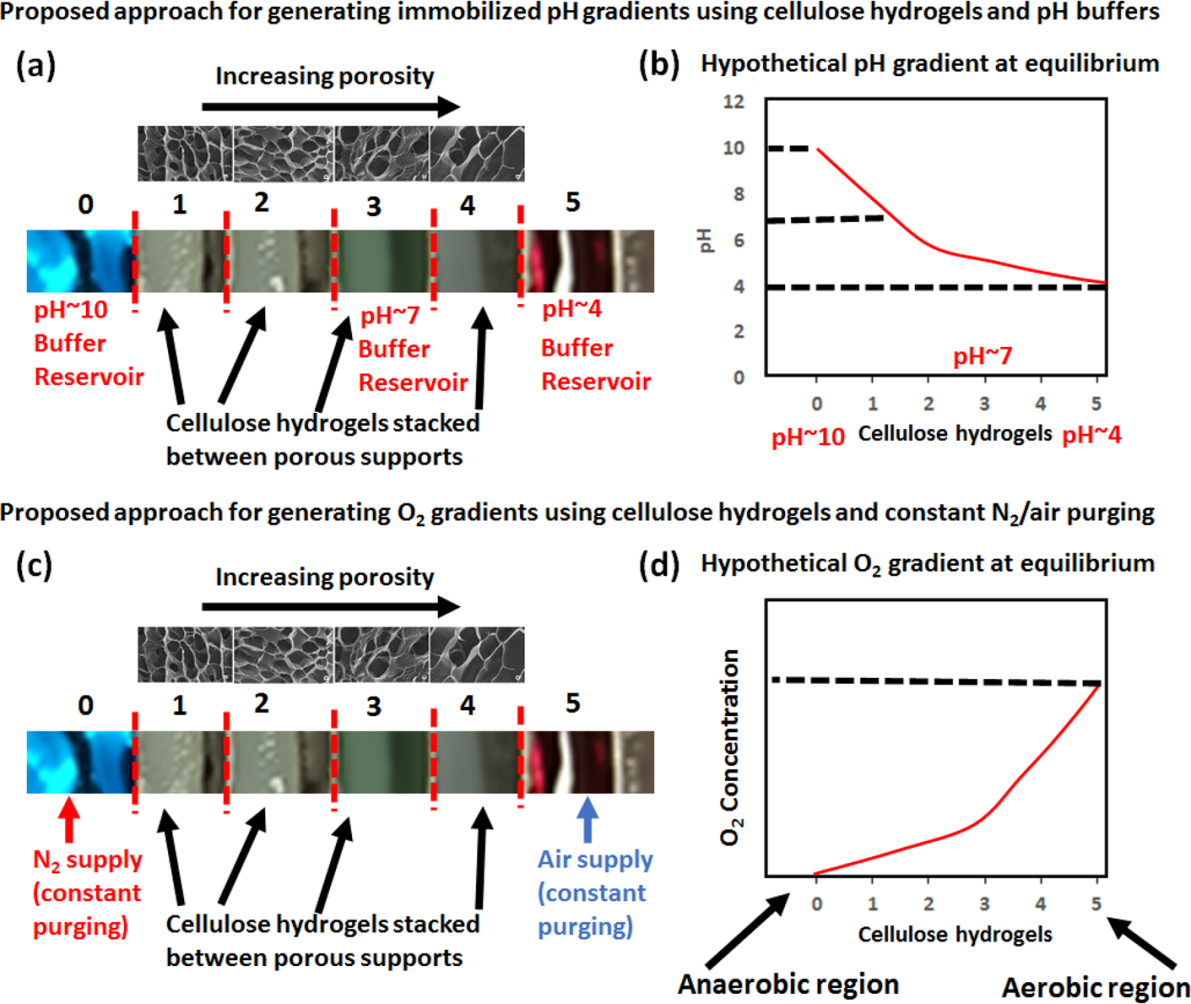
Proposed approaches on creating pH and O_2_ gradients using cellulose hydrogels, pH buffers, nitrogen (N_2_), and air supply.

As shown in Figure 5, the proposed approach uses stacking of cellulose hydrogels of varying porosity separated by porous supports with a glued porous membrane. The porous supports separate the hydrogels from each other by porous membranes that allow for the bacteria growth media and buffers to freely flow between the hydrogels. Low-porosity hydrogels will be placed close to the high pH buffer reservoir to simulate the rectum region of the human gut, whereas highly porous hydrogels are stacked close to low pH buffer to simulate the stomach region (Figure 5a). If needed, a neutral pH buffer can be placed at the center of the hydrogel stack. The establishment of pH gradients at room temperature may take a long time to achieve as a result of slow diffusion of buffers through cellulose hydrogels. Generally, the hydrogel bioreactor is filled with media for bacteria growth and autoclaved at 121 °C for 15 min. The autoclaving procedure allows for the rapid establishment of pH gradients as a result of increased diffusion of buffers at a high temperature. We show a hypothetical equilibrium pH gradient that can be achieved using our proposed approach (Figure 5b).

Similarly, oxygen gradients can be achieved using the same hydrogel stacking method. To establish oxygen gradients on top of the previously established pH gradient within the hydrogel bioreactor, a high pH buffer simulating the rectum region will be constantly purged with pure nitrogen (N_2_) and low pH buffer with air (Figure 5c). The flow rates of nitrogen and air supply and hydrogel porosity tuning can be used to tune the slope of oxygen gradients. We show a hypothetical equilibrium oxygen gradient that can be achieved using our proposed approach (Figure 5d).

## Use of a Cellulose-Hydrogel-Based Gut-Like Bioreactor for Multi-strain Probiotic Production

**Figure 6:**
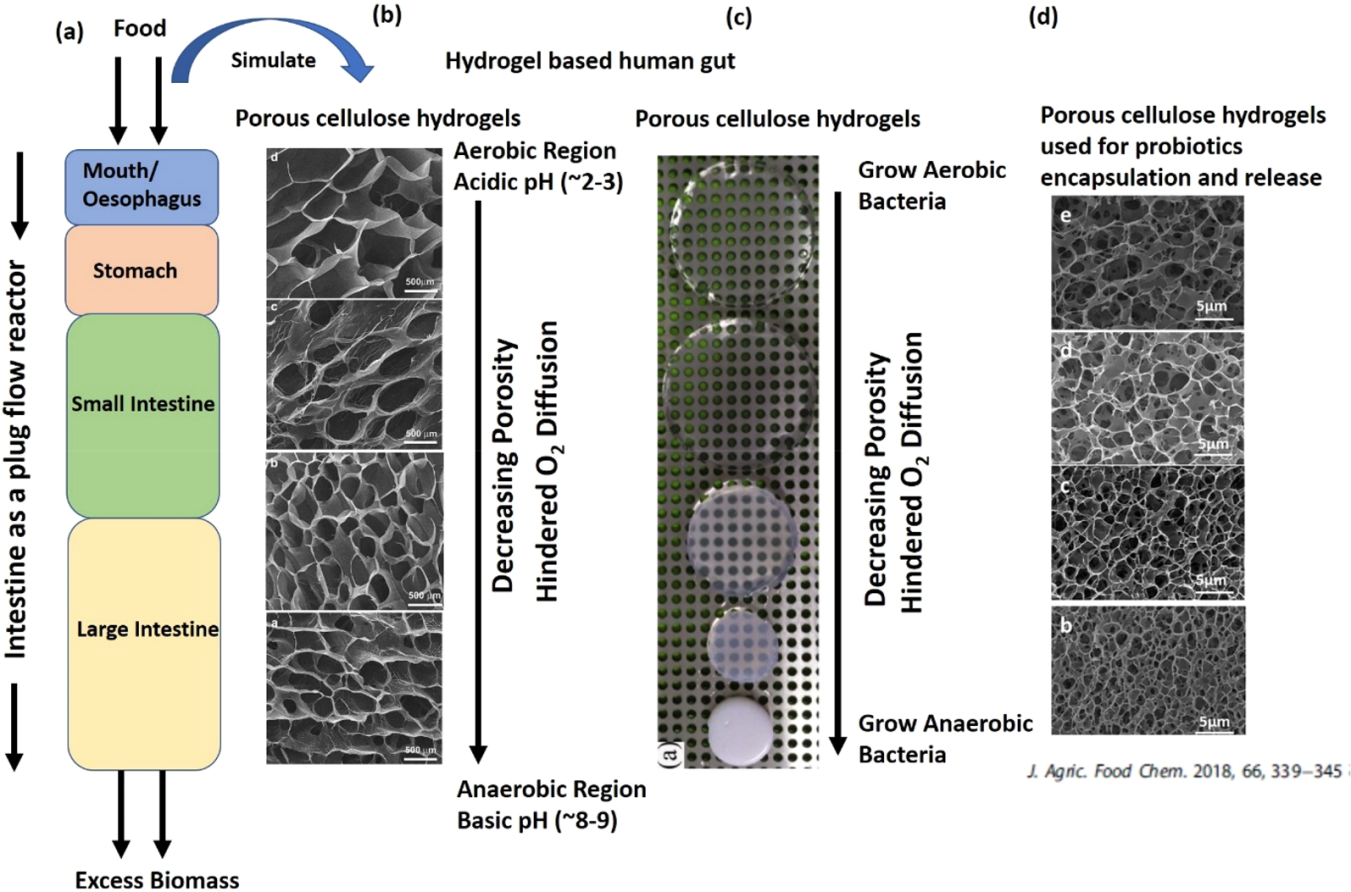
Human gut as a compartmentalized plug flow reactor, with simulation of the gut using porous hydrogels to maintain pH and oxygen gradients as maintained across the digestive tract.^52^ Examples of controlled porosity hydrogels synthesized by various researchers are presented as examples.^26,42,46^ Controlled porosity cellulose-based hydrogels have already been used by ref (26) for encapsulation and release of probiotic bacteria.

**Figure 7:**
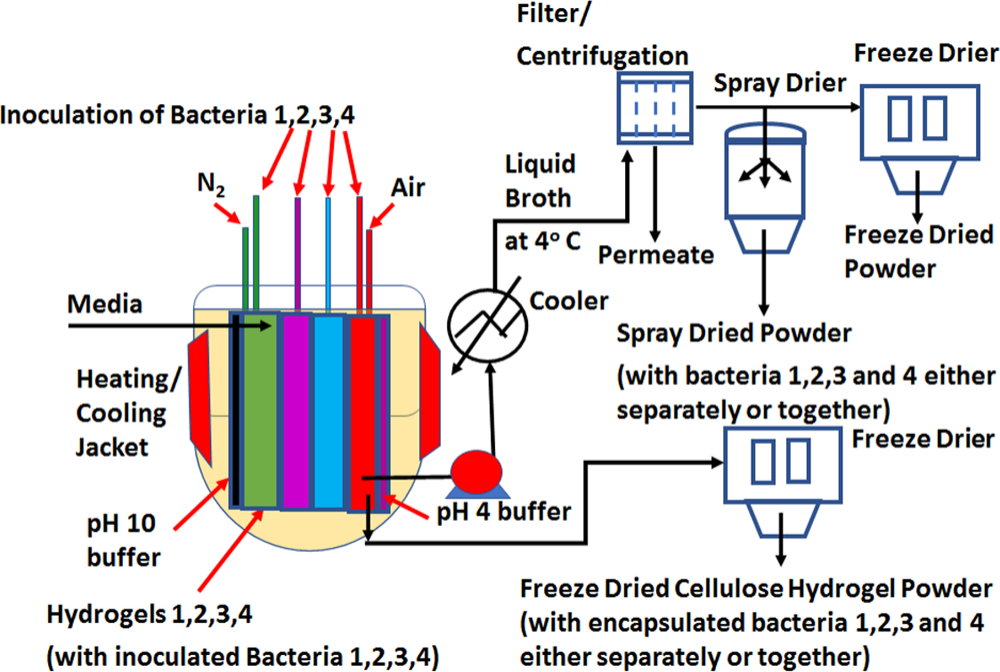
Proposed multi-strain aerobic/anaerobic probiotic bacteria production by a multiple cellulose-hydrogel-based gut-like bioreactor and possible harvesting procedure of liquid broth separately from immobilized bacteria on cellulose hydrogels. The freeze-dried cellulose powder will encapsulate the immobilized bacteria on the hydrogels.

As mentioned earlier, from a chemical engineering point of view, the gut can be treated as a compartmentalized plug flow reactor (Figure 6).^52^ Although it is very simplified compared to complexity that exists in the human gut, such a simplification and idealization of regions makes it easy to design a hydrogel-based bioreactor. In this proposed idealized bioreactor filled with various cellulose hydrogels in various interconnected compartments that can have crosstalk between them as in the human gut, such an arrangement has the potential to simulate realistic gastrointestinal conditions in the bioreactor. Once the control of the pH and oxygen gradients are achieved as schematically shown in Figure 7, the bioreactor can be inoculated with a mixture of aerobic and anaerobic gut microbes. The inoculation of individual strains in different parts of the reactor with a favorable growth environment is one possible option. The second method is to inoculate multiple strains together as a mixture in each interconnected compartment of the bioreactor. The microbes might colonize and grow in their comfort zones of a favorable environment, including pH and pO_2_ values (Figures 6 and 7).

## Proposed Harvesting Approaches for Extraction of Either Individual or a Mixture of Probiotics from a Gut-Like Cellulose-Based Hydrogel Bioreactor

In conventional liquid media-based bioreactors, the single strain of probiotic bacteria grown is generally separated from liquid broth using either membrane filtration or centrifugation. However, in the case of the proposed cellulose-hydrogel-based gut-like bioreactor, the harvesting of multiple probiotic bacteria grown can be achieved in two ways, as schematically shown in Figure 7. As the individual probiotic bacteria are grown in their own specific hydrogel compartment, each bacteria can be harvested either separately or the consortia of bacteria can be extracted together. As the bacteria grows in the liquid media surrounding each hydrogel as well as immobilizes on the hydrogel, they can be harvested separately. In the first case of bacteria harvest from liquid broth, it follows conventional harvesting using filtration/centrifugation, followed by spray or freeze drying. In the second case of harvesting bacteria immobilized on cellulose hydrogels, the whole hydrogel can be freeze-dried directly. In this case, the hydrogel-immobilized bacteria become encapsulated within the cellulose hydrogel matrix that can potentially protect them from a harsh gastric environment.

The third option is to freeze dry filtered liquid broth along with the hydrogels, so that bacteria in liquid media as well as immobilized bacteria become encapsulated within the cellulose hydrogel matrix. The third option has the potential to reduce the cost of production because the harvesting and encapsulation steps are combined into one step. As discussed in the next section, cellulose-based hydrogels have shown great promise as encapsulation material for probiotic bacteria.

## Use of Cellulose-Based Hydrogels for Probiotic Encapsulation

**Figure 8:**
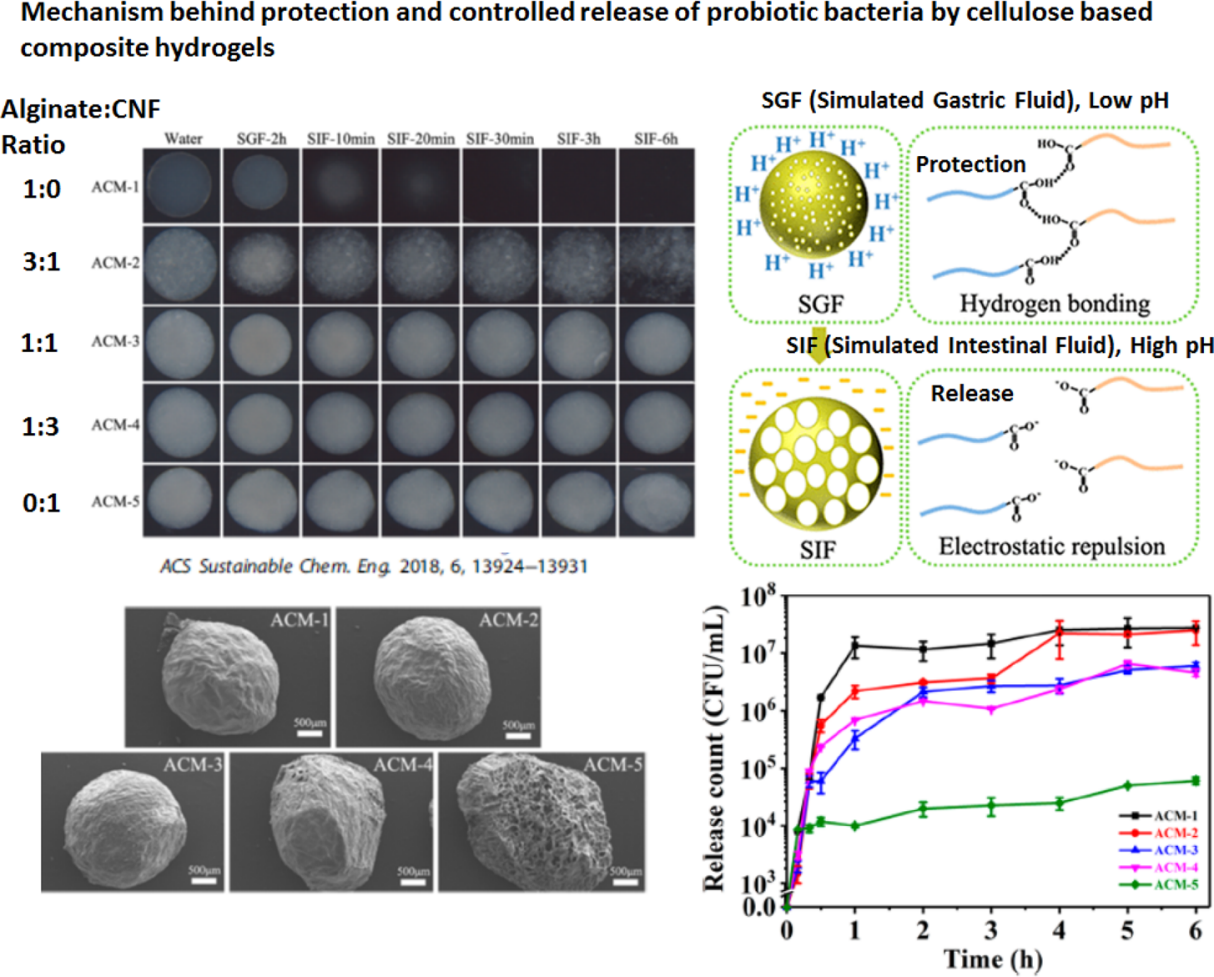
Mechanism behind the protection of probiotic bacteria by cellulose composite hydrogels. The shrinkage of the composite hydrogel network as a result of protonation protects the probiotics in low pH (stomach), whereas the swelling in high pH (intestine) releases the probiotic bacteria.^31^ This figure was redrawn and adapted with permission from ref (31). Copyright 2018 American Chemical Society (ACS).

As mentioned in the previous section, when multiple-strain probiotics immobilized on cellulose hydrogel of various porosities are freeze-dried, the cellulose hydrogels can encapsulate the probiotics. Before we discuss how various cellulose hydrogels can be used for controlled delivery (Figure 8), we provide recent progress in this area. It is well-established that selection of suitable encapsulating material is of prime importance to ensure the effectiveness of probiotics. Over the last decades, studies have been conducted on development of novel and efficient biocomposites for possible application in probiotic encapsulation.^75,76,13,77−81^ There have been excellent reviews published in this area; hence, for the sake of brevity and considering the scope of this perspective, we refer the reader to the recent reviews.^82−85^ Despite progress made in the field, novel approaches toward the encapsulation of probiotics ensuring high viability, efficiency, biocompatibility, and timely and targeted release of probiotic cells represent a field of opportunities that need to be explored.

## Recent Advances in Probiotic Encapsulation: Use of Cellulose Hydrogels

Cellulose-based hydrogels are promising material that allow for release of probiotic bacteria into the intestine. Both hydroxypropyl methoxy cellulose (HPMC) and carboxymethyl cellulose (CMC) have been reported to offer better protection and stabilization against adverse GI conditions.^15^Table 1 describes the applications of cellulose hydrogels for encapsulation of probiotic bacteria.

**Table 1:** Application of Cellulose-Based Composite Hydrogels for Encapsualtion and Controlled Release of Probiotic Bacteria

year	cellulose type	seconday carbohydrate polymer	cross-linking agent	probiotic bacteria encapsulated	reference
2011	carboxymethyl cellulose (CMC)	chitosan	layer by layer	*Lactobacillus acidophilus*	(23)
2016	bacterial cellulose (BC)	none	adsorption–incubation/co-culturing with BC producing *Gluconacetobacter xylinus* bacteria	*Lactobacillus delbrueckii* PKM 490, *Lactobacillus plantarum* DSM 13273, and *Lactobacillus casei* ATCC 393	(86)
2016	regenerated cellulose from cotton pulp (RC)	sodium alginate	sol–gel transition	*Lactobacillus plantarum* (LP)	(32)
2017	cellulose nanocrystals (CNCs)	sodium alginate	CaCl_2_	*Lactobacillus rhamnosus* (ATCC 9595)	(87)
2017–2018	CMC	chitosan	genipin	*Lactobacillus rhamnosus* GG (LGG)	(28 and 30)
2017	RC	sodium alginate (as a housing)	CaCl_2_	*Lactobacillus plantarum* (LP)	(33)
2017	CMC	κ-carrageenan	blends	*Lactobacillus plantarum* (LP)	(29)
2017	CNF–TEMPO (TEMPO-oxidized cellulose nanofiber)	cellulose fiber (CF)	hydrochloric acid	*Lactobacillus plantarum* (LP)	(26)
2018	CNF–TEMPO	sodium alginate	CaCl_2_	*Lactobacillus plantarum* (LP)	(31)
2019	CMC	hydroxyethyl cellulose (HEC)	citric acid	*Lactobacillus rhamnosus* GG (LGG)	(27)
2019	CMC, MC, and HPMC (methyl cellulose and hydroxypropyl methoxy cellulose)	gum arabic/skim milk	blends	*Lactobacillus paracasei* strain Lpc-37	(15)

As shown in Table 1, cellulose hydrogel composites either using a combination of cellulosic materials [cellulose fiber (CF), cellulose nanofiber (CNF), cellulose nanocrystals (CNCs), carboxymethyl cellulose (CMC), methyl cellulose (MC), hydroxypropyl methoxy cellulose (HPMC), and hydroxyethyl cellulose (HEC)] or other biopolymers [sodium alginate (SA), gum arabic, chitosan, and κ-carrageenan] are successfully used for encapsulation of probiotic bacteria for colonic delivery. It has been shown that cellulose hydrogels protect the probiotic bacteria in simulated gastric fluid (SGF) but slowly released in simulated intestinal fluid (SIF) for colonic delivery.

We describe two important studies by Zhang et al.^31^ and Luan et al.,^26^ where a combination of alginate/CNF and native CF/CNF were used to control the porosity, water absorption, and swelling of the hydrogel network in various pH conditions. Hao et al.^31^ used alginate/CNF in various ratios to prepare hydrogels of various transparencies and porosities, as shown in Figure 8. They found that alginate-only hydrogels (ACM-1 in Figure 8) completely dissolve within 20 min of immersion in SIF, whereas hydrogels made with a 1:1 ratio of alginate/CNF survive in SIF for 6 h. As shown in Figure 8, the probiotic bacteria were released within 2 h of immersion in SIF in the case of highly porous hydrogels, whereas in the case of low-porosity hydrogels, controlled release of up to 6 h was observed.

Zhang et al.^31^ presented a mechanism of protection of probiotic bacteria by SA–CNF composite hydrogels, as schematically shown in Figure 8. Zhang et al.^31^ explained that, in acidic pH, protonation of carboxylic acid groups in sodium alginate and CNF chains contributes to reduced electrostatic repulsion.^31^ The increased attraction results in the formation of hydrogen bonds between carboxylic acid and protonated carboxyl groups. This leads to the shrinkage of the SA–CNF composite hydrogel and protection of probiotic bacteria being released in the stomach.^31^ In acidic pH, the shrinkage was found to decrease with the increase in cellulose concentration SA–CNF composite hydrogels, whereas pure CNF hydrogel did not shrink. However, in basic pH, SA–CNF composite hydrogels swelled, whereas CNF hydrogels did not swell. In both acidic and basic pH cases, the CNF backbone imparted stability to the structure of composite hydrogels.^31^ Such a protonation and deprotonation mechanism in acidic and basic pH conditions could be the reason for probiotic encapsulation and controlled release properties of cellulose hydrogels.^26,31^

**Figure 9:**
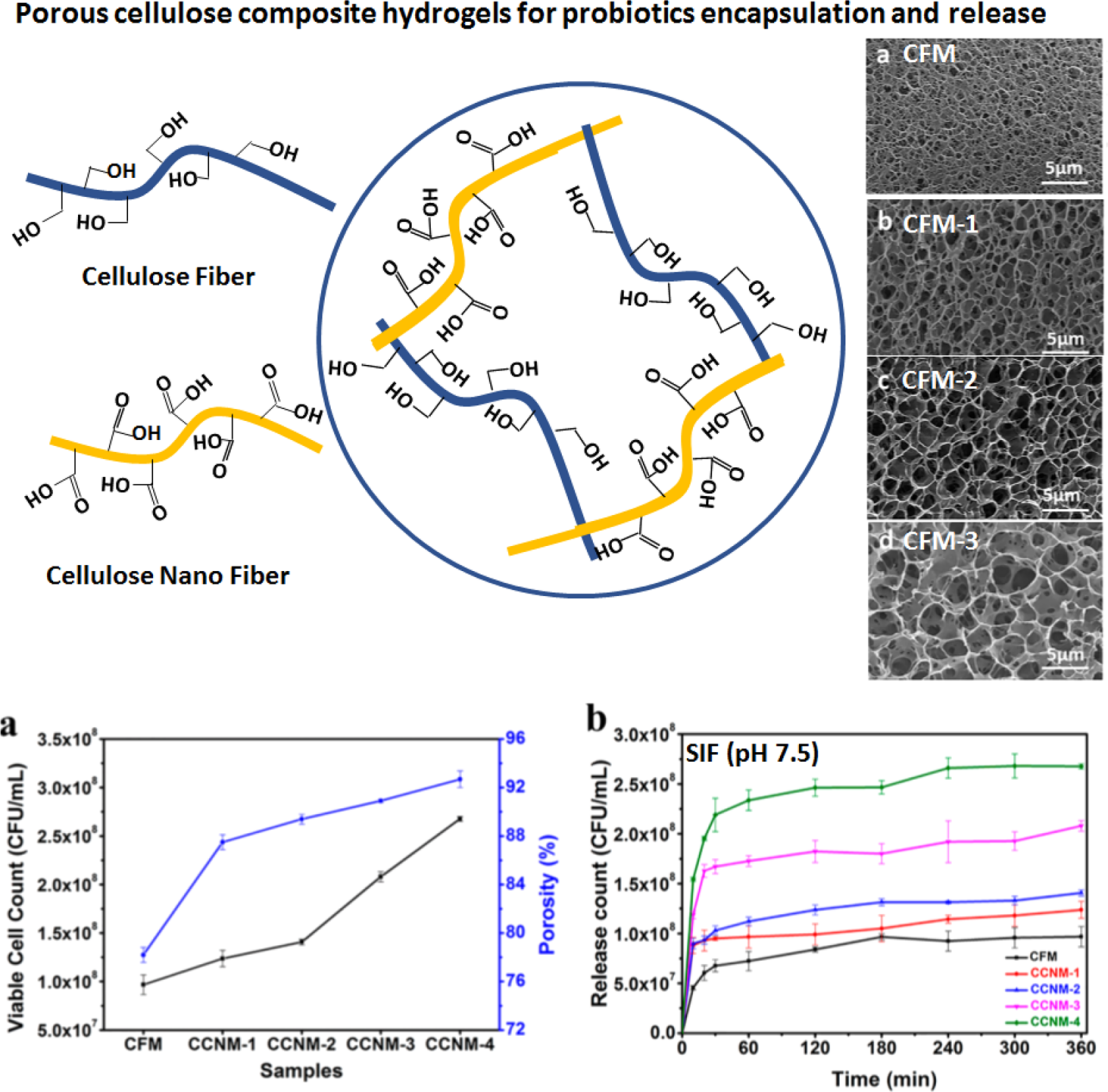
Controlled porosity cellulose composite hydrogels for probiotic encapsulation and release. The porosity increased with the increase in CNF in cellulose fiber/cellulose nanofiber (CF/CNF) composite hydrogels that controlled the release of viable cells of probiotic bacteria. This figure was redrawn and adapted with permission from ref (26). Copyright 2018 American Chemical Society. The shrinkage and swelling depend upon the carboxylic group content and the porosity or density of the network that protects the probiotic bacteria.^26^

As described earlier, Luan et al.^26^ used a combination of native CF and CNF to prepare controlled porosity cellulose macrogel particles, where it was found that, with an increase in the CNF concentration, the average porosity increased from 0.6 to 2.1 μm. As shown in Figure 9, the probiotic bacteria were released within 2–3 h of immersion in SIF in the case of highly porous hydrogels, whereas in the case of low-porosity hydrogels, controlled release of up to 6 h was observed. In this case of cellulose-only composite hydrogels as opposed to the SA–CNF case presented above, the shrinkage and swelling of the hydrogel networks depend upon the carboxylic content of the material.^26^

In the case of SA–CNF composite hydrogels, Zhang et al.^31^ found that there was an ionic interaction and cross-linking between adjacent SA and the carboxyl group of CNF molecules. In this case, both SA and carboxyl contents of CNF provided the required pH responsive behavior. The CNF network also provided overall structural stability required to withstand dissolution in SIF. However, in the case of the CF–CNF composite hydrogel, Luan et al.^26^ have found that pH-responsive behavior was imported to the hydrogels by the carboxyl content of CNF. The porosity also correlated well with the carboxyl content of CNF in the composite hydrogel. In acidic pH (SGF), the CF–CNF hydrogel with a higher amount CNF (high porosity) shrank a little but swelled a lot in basic pH (SIF). Hence, the porosity as well as swelling behavior resulting from the carboxyl content of CNF contributed to the release of encapsulated probiotic bacteria. Highly porous hydrogels swell a lot in SIF and release the encapsulated probiotics quickly, whereas low-porosity hydrogels with high amounts CF do not swell much in SIF and, hence, release the bacteria over time.

## Proposed Approach in Controlled Delivery of Multi-stain Probiotics Using Cellulose Hydrogels

As explained in the previous section by two example studies of Zhang et al.^31^ and Luan et al.,^26^ composite cellulose hydrogels can be used to control the release of probiotic bacteria by tuning either the dissolution of one component in the hydrogel or the porosity. These studies form the basis for our proposed approach in using a cellulose hydrogel bioreactor for encapsulation and controlled release of multiple probiotic bacteria.

As explained earlier, probiotic bacteria that favor low pH and aerobic conditions for growth will be grown in highly porous hydrogels, whereas probiotic bacteria that favor high pH and strictly anaerobic conditions will be grown in low-porous hydrogels. When these individual hydrogels with individual probiotic bacteria are freeze-dried, the bacteria become encapsulated within the respective hydrogel matrix. During the transit through the GI tract, the bacteria encapsulated in highly porous hydrogels are released first, followed by less porous hydrogels. When the porosity and pH responsiveness of the hydrogels are tuned, the controlled release of individual bacteria in the region of the gut favorable for the respective probiotic bacteria can be achieved.

Fermentation strategies, such as immobilized cellulose-hydrogel-based bioreactors, are highly suitable and promising to produce multiple probiotic strains. With the ability to maintain pH gradients and diffused O_2_ levels, hydrogel-based bioreactors and immobilization technology appear to be a feasible option.

Additionally, on the basis of the stability, biosustainability, and biodegradability of cellulose nanofibers, application of cellulose hydrogels in the fermenter can propel the research on designing probiotics in a whole new dimension. Designing such a study where probiotics are propagating in their natural habitat (or closest to what is found in the human intestinal tract) requires elaborated levels of planning and engineering. However, such studies are crucial considering the present scenario because the hydrogels are non-toxic, non-hazardous, recyclable, and biodegradable. Additionally, incorporation of these hydrogels will also lead the way forward for attaining sustainable development goals and achieving a circular economy. There are already a few studies that implemented the idea of cultivating more than two probiotic strains using the co-culture fermentation method in a bioreactor.^88^ Additionally, there are reports on the application of the continuous production strategy for large-scale cultivation of probiotics. Although the strategy has been quite successful for providing a high cell yield, continuous fermentation always runs a risk of contamination and, even more importantly, altering the characteristics of probiotics in the process.^89^ Nonetheless, over the past few years, the concept of immobilization has been applied for a great length in probiotic research. The commercial production of probiotics and especially monoculture probiotics is still largely based on the free cell culture in batch mode. This is primarily due to the fact that bacterial cells produced through the process of immobilization vary greatly in terms of physiology, morphology, and growth characteristics.^90^ Future research should be directed toward efficient immobilization support for large-scale production of food items, such as yogurt, milk solids, and cornflakes.^91^

This perspective proposes the concept of designing next-generation bioreactors with the central idea of applying cellulose hydrogels for efficient production of multiple probiotic strains using a conventional laboratory-scale fermenter. As described earlier in the perspective, properties of cellulose hydrogels make it feasible to control and set up pH and pO_2_ gradients. Essentially, the prospects hint toward emerging engineering aspects along with simulation studies of the intestinal tract for efficient probiotic production along with generating a deeper understanding of the human gut ecosystem.

## References

[ref1] O’Toole P. W.; Marchesi J. R.; Hill C. Next-generation probiotics: The spectrum from probiotics to live biotherapeutics. Nat. Microbiol. 2017, 2 (5), 17057 10.1038/nmicrobiol.2017.57. 28440276

[ref2] Zendeboodi F.; Khorshidian N.; Mortazavian A. M.; da Cruz A. G. Probiotic: Conceptualization from a new approach. Curr. Opin. Food Sci. 2020, 32, 103–123. 10.1016/j.cofs.2020.03.009.

[ref3] Szajewska H.; Canani R. B.; Guarino A.; Hojsak I.; Indrio F.; Kolacek S.; Orel R.; Shamir R.; Vandenplas Y.; van Goudoever J. B.; Weizman Z. Probiotics for the prevention of antibiotic-associated diarrhea in children. J. Pediatr. Gastroenterol. Nutr. 2016, 62 (3), 495–506. 10.1097/MPG.0000000000001081. 26756877

[ref4] Piqué N.; Berlanga M.; Miñana-Galbis D. Health benefits of heat-killed (Tyndallized) probiotics: An overview. Int. J. Mol. Sci. 2019, 20 (10), 2534 10.3390/ijms20102534. PMC6566317 31126033

[ref5] Anal A. K.; Singh H. Recent advances in microencapsulation of probiotics for industrial applications and targeted delivery. Trends Food Sci. Technol. 2007, 18 (5), 240–251. 10.1016/j.tifs.2007.01.004.

[ref6] Terpou A.; Papadaki A.; Lappa I. K.; Kachrimanidou V.; Bosnea L. A.; Kopsahelis N. Probiotics in food systems: Significance and emerging strategies towards improved viability and delivery of enhanced beneficial value. Nutrients 2019, 11 (7), 1591 10.3390/nu11071591. PMC6683253 31337060

[ref7] Papadimitriou K.; Zoumpopoulou G.; Foligné B.; Alexandraki V.; Kazou M.; Pot B.; Tsakalidou E. Discovering probiotic microorganisms: In vitro, in vivo, genetic and omics approaches. Front. Microbiol. 2015, 6, 58 10.3389/fmicb.2015.00058. 25741323 PMC4330916

[ref8] Vera-Pingitore E.; Jimenez M. E.; Dallagnol A.; Belfiore C.; Fontana C.; Fontana P.; von Wright A.; Vignolo G.; Plumed-Ferrer C. Screening and characterization of potential probiotic and starter bacteria for plant fermentations. LWT—Food Sci. Technol. 2016, 71, 288–294. 10.1016/j.lwt.2016.03.046.

[ref9] Mollakhalili M. N.; Mortazavian A. M.; Sohrabvandi S.; da Cruz A. G.; Mohammadi R. Probiotic supplements and food products: Comparison for different targets. Appl. Food Biotechnol. 2017, 4 (3), 123–132. 10.22037/afb.v4i3.16420.

[ref10] Kechagia M.; Basoulis D.; Konstantopoulou S.; Dimitriadi D.; Gyftopoulou K.; Skarmoutsou N.; Fakiri E. M. Health benefits of probiotics: A review. ISRN Nutr. 2013, 2013, 1–7. 10.5402/2013/481651. PMC4045285 24959545

[ref11] Majeed M.; Majeed S.; Nagabhushanam K.; Arumugam S.; Beede K.; Ali F. Evaluation of the in vitro cholesterol-lowering activity of the probiotic strain Bacillus coagulans MTCC 5856. Int. J. Food Sci. Technol. 2019, 54 (1), 212–220. 10.1111/ijfs.13926.

[ref12] Puniya M.; Ravinder Kumar M.; Panwar H.; Kumar N.; Ramneek A. K. P. Screening of lactic acid bacteria of different origin for their probiotic potential. J. Food Process. Technol. 2016, 7 (1), 545 10.4172/2157-7110.1000545.

[ref13] González-Ferrero C.; Irache J. M.; Marín-Calvo B.; Ortiz-Romero L.; Virto-Resano R.; González-Navarro C. J. Encapsulation of probiotics in soybean protein-based microparticles preserves viable cell concentration in foods all along the production and storage processes. J. Microencapsulation 2020, 37 (3), 242–253. 10.1080/02652048.2020.1724203. 31997685

[ref14] Hernández-Barrueta T.; Martínez-Bustos F.; Castaño-Tostado E.; Lee Y.; Miller M. J.; Amaya-Llano S. L. Encapsulation of probiotics in whey protein isolate and modified huauzontle’s starch: An approach to avoid fermentation and stabilize polyphenol compounds in a ready-to-drink probiotic green tea. LWT 2020, 124, 109131 10.1016/j.lwt.2020.109131.

[ref15] Tao T.; Ding Z.; Hou D.; Prakash S.; Zhao Y.; Fan Z.; Zhang D.; Wang Z.; Liu M.; Han J. Influence of polysaccharide as co-encapsulant on powder characteristics, survival and viability of microencapsulated Lactobacillus paracasei Lpc-37 by spray drying. J. Food Eng. 2019, 252, 10–17. 10.1016/j.jfoodeng.2019.02.009.

[ref16] Tang D. Y. Y.; Khoo K. S.; Chew K. W.; Tao Y.; Ho S.-H.; Show P. L. Potential utilization of bioproducts from microalgae for the quality enhancement of natural products. Bioresour. Technol. 2020, 304, 122997 10.1016/j.biortech.2020.122997. 32094007

[ref17] Hassan S. S.; Fadzil I. N. A.; Yusoff A.; Khalil K. A. A Review on Microencapsulation in Improving Probiotic Stability for Beverages Application. Sci. Lett. 2020, 14 (1), 49–58. 10.1234/jmpc.v14i1.7782.

[ref18] Nickerson M.; Low N.; Korber D.; Wang J.; Khan N. Microcapsules containing probiotics and methods of making same. WO Patent 2015019307 A1, Feb 12, 2015.

[ref19] Amin T.; Thakur M.; Jain S. Microencapsulation—The future of probiotic cultures. J. Microbiol., Biotechnol. Food Sci. 2020, 9 (4), 35–43.

[ref20] Raise A.; Dupont S.; Iaconelli C.; Caliri C.; Charriau A.; Gervais P.; Chambin O.; Beney L. Comparison of two encapsulation processes to protect the commensal gut probiotic bacterium Faecalibacterium prausnitzii from the digestive tract. J. Drug Delivery Sci. Technol. 2020, 56, 101608 10.1016/j.jddst.2020.101608.

[ref21] Li W.; Liu L.; Tian H.; Luo X.; Liu S. Encapsulation of Lactobacillus plantarum in cellulose based microgel with controlled release behavior and increased long-term storage stability. Carbohydr. Polym. 2019, 223, 115065 10.1016/j.carbpol.2019.115065. 31426953

[ref22] Sultana K.; Godward G.; Reynolds N.; Arumugaswamy R.; Peiris P.; Kailasapathy K. Encapsulation of probiotic bacteria with alginate-starch and evaluation of survival in simulated gastrointestinal conditions and in yoghurt. Int. J. Food Microbiol. 2000, 62 (1), 47–55. 10.1016/S0168-1605(00)00380-9. 11139021

[ref23] Priya A. J.; Vijayalakshmi S. P.; Raichur A. M. Enhanced Survival of Probiotic Lactobacillus acidophilus by Encapsulation with Nanostructured Polyelectrolyte Layers through Layer-by-Layer Approach. J. Agric. Food Chem. 2011, 59 (21), 11838–11845. 10.1021/jf203378s. 21958340

[ref24] Ramos P. E.; Cerqueira M. A.; Teixeira J. A.; Vicente A. A. Physiological protection of probiotic microcapsules by coatings. Crit. Rev. Food Sci. Nutr. 2018, 58 (11), 1864–1877. 10.1080/10408398.2017.1289148. 28362165

[ref25] Hansen L. T.; Allan-Wojtas P. M.; Jin Y. L.; Paulson A. T. Survival of Ca-alginate microencapsulated Bifidobacterium spp. in milk and simulated gastrointestinal conditions. Food Microbiol. 2002, 19 (1), 35–45. 10.1006/fmic.2001.0452.

[ref26] Luan Q.; Zhou W.; Zhang H.; Bao Y.; Zheng M.; Shi J.; Tang H.; Huang F. Cellulose-based composite macrogels from cellulose fiber and cellulose nanofiber as intestine delivery vehicles for probiotics. J. Agric. Food Chem. 2018, 66 (1), 339–345. 10.1021/acs.jafc.7b04754. 29224351

[ref27] Singh P.; Magalhães S.; Alves L.; Antunes F.; Miguel M.; Lindman B.; Medronho B. Cellulose-based edible films for probiotic entrapment. Food Hydrocolloids 2019, 88, 68–74. 10.1016/j.foodhyd.2018.08.057.

[ref28] Singh P.; Medronho B.; Alves L.; da Silva G. J.; Miguel M. G.; Lindman B. Development of carboxymethyl cellulose-chitosan hybrid micro- and macroparticles for encapsulation of probiotic bacteria. Carbohydr. Polym. 2017, 175, 87–95. 10.1016/j.carbpol.2017.06.119. 28917929

[ref29] Dafe A.; Etemadi H.; Zarredar H.; Mahdavinia G. R. Development of novel carboxymethyl cellulose/k-carrageenan blends as an enteric delivery vehicle for probiotic bacteria. Int. J. Biol. Macromol. 2017, 97, 299–307. 10.1016/j.ijbiomac.2017.01.016. 28064052

[ref30] Singh P.; Medronho B.; Santos T. d.; Nunes-Correia I.; Granja P.; Miguel M. G.; Lindman B. On the viability, cytotoxicity and stability of probiotic bacteria entrapped in cellulose-based particles. Food Hydrocolloids 2018, 82, 457–465. 10.1016/j.foodhyd.2018.04.027.

[ref31] Zhang H.; Yang C.; Zhou W.; Luan Q.; Li W.; Deng Q.; Dong X.; Tang H.; Huang F. A pH-Responsive Gel Macrosphere Based on Sodium Alginate and Cellulose Nanofiber for Potential Intestinal Delivery of Probiotics. ACS Sustainable Chem. Eng. 2018, 6 (11), 13924–13931. 10.1021/acssuschemeng.8b02237.

[ref32] Li W.; Luo X.; Song R.; Zhu Y.; Li B.; Liu S. Porous Cellulose Microgel Particle: A Fascinating Host for the Encapsulation, Protection, and Delivery of Lactobacillus plantarum. J. Agric. Food Chem. 2016, 64 (17), 3430–3436. 10.1021/acs.jafc.6b00481. 27068772

[ref33] Li W.; Zhu Y.; Ye F.; Li B.; Luo X.; Liu S. Probiotics in cellulose houses: Enhanced viability and targeted delivery of Lactobacillus plantarum. Food Hydrocolloids 2017, 62, 66–72. 10.1016/j.foodhyd.2016.07.019.

[ref34] Bill and Melinda Gates Foundation. New Approaches for Manufacturing Gut Microbial Biotherapeutics (Round 22); https://gcgh.grandchallenges.org/challenge/new-approaches-manufacturing-gut-microbial-biotherapeutics-round-22 (accessed July 22, 2020).

[ref35] Blanton L. V.; Barratt M. J.; Charbonneau M. R.; Ahmed T.; Gordon J. I. Childhood undernutrition, the gut microbiota, and microbiota-directed therapeutics. Science 2016, 352 (6293), 1533–1533. 10.1126/science.aad9359. 27339978

[ref36] Costello E. K.; Relman D. A. Immaturity in the gut microbial community. Nature 2014, 510 (7505), 344–345. 10.1038/nature13347. 24896185

[ref37] Gehrig J. L.; Venkatesh S.; Chang H.-W.; Hibberd M. C.; Kung V. L.; Cheng J.; Chen R. Y.; Subramanian S.; Cowardin C. A.; Meier M. F.; O’Donnell D.; Talcott M.; Spears L. D.; Semenkovich C. F.; Henrissat B.; Giannone R. J.; Hettich R. L.; Ilkayeva O.; Muehlbauer M.; Newgard C. B.; Sawyer C.; Head R. D.; Rodionov D. A.; Arzamasov A. A.; Leyn S. A.; Osterman A. L.; Hossain M. I.; Islam M.; Choudhury N.; Sarker S. A.; Huq S.; Mahmud I.; Mostafa I.; Mahfuz M.; Barratt M. J.; Ahmed T.; Gordon J. I. Effects of microbiota-directed foods in gnotobiotic animals and undernourished children. Science 2019, 365 (6449), 4732 10.1126/science.aau4732. PMC6683325 31296738

[ref38] Pennisi E. Gut microbes may help malnourished children. Science 2019, 365 (6449), 109 10.1126/science.365.6449.109. 31296748

[ref39] Raman A. S.; Gehrig J. L.; Venkatesh S.; Chang H.-W.; Hibberd M. C.; Subramanian S.; Kang G.; Bessong P. O.; Lima A. A.M.; Kosek M. N.; Petri W. A.; Rodionov D. A.; Arzamasov A. A.; Leyn S. A.; Osterman A. L.; Huq S.; Mostafa I.; Islam M.; Mahfuz M.; Haque R.; Ahmed T.; Barratt M. J.; Gordon J. I. A sparse covarying unit that describes healthy and impaired human gut microbiota development. Science 2019, 365 (6449), 4735 10.1126/science.aau4735. PMC6683326 31296739

[ref40] Subramanian S.; Huq S.; Yatsunenko T.; Haque R.; Mahfuz M.; Alam M. A.; Benezra A.; DeStefano J.; Meier M. F.; Muegge B. D.; Barratt M. J.; VanArendonk L. G.; Zhang Q.; Province M. A.; Petri W. A. Jr; Ahmed T.; Gordon J. I. Persistent gut microbiota immaturity in malnourished Bangladeshi children. Nature 2014, 510 (7505), 417–421. 10.1038/nature13421. 24896187 PMC4189846

[ref41] Chang C.; Duan B.; Cai J.; Zhang L. Superabsorbent hydrogels based on cellulose for smart swelling and controllable delivery. Eur. Polym. J. 2010, 46 (1), 92–100. 10.1016/j.eurpolymj.2009.04.033.

[ref42] Chang C.; Duan B.; Zhang L. Fabrication and characterization of novel macroporous cellulose-alginate hydrogels. Polymer 2009, 50 (23), 5467–5473. 10.1016/j.polymer.2009.06.001.

[ref43] Chang C.; Lue A.; Zhang L. Effects of Crosslinking Methods on Structure and Properties of Cellulose/PVA Hydrogels. Macromol. Chem. Phys. 2008, 209 (12), 1266–1273. 10.1002/macp.200800161.

[ref44] Chang C.; Zhang L. Cellulose-based hydrogels: Present status and application prospects. Carbohydr. Polym. 2011, 84 (1), 40–53. 10.1016/j.carbpol.2010.12.023.

[ref45] Chang C.; Zhang L.; Zhou J.; Zhang L.; Kennedy J. F. Structure and properties of hydrogels prepared from cellulose in NaOH/urea aqueous solutions. Carbohydr. Polym. 2010, 82 (1), 122–127. 10.1016/j.carbpol.2010.04.033.

[ref46] Geng H. A one-step approach to make cellulose-based hydrogels of various transparency and swelling degrees. Carbohydr. Polym. 2018, 186, 208–216. 10.1016/j.carbpol.2018.01.031. 29455980

[ref47] Geng H. A facile approach to light weight, high porosity cellulose aerogels. Int. J. Biol. Macromol. 2018, 118, 921–931. 10.1016/j.ijbiomac.2018.06.167. 29964109

[ref48] Bugs in Your Guts. What Is the Human Gut Microbiota? http://bugs-in-your-guts.com/?cat=6 (accessed July 22, 2020).

[ref49] Espey M. G. Role of oxygen gradients in shaping redox relationships between the human intestine and its microbiota. Free Radical Biol. Med. 2013, 55, 130–140. 10.1016/j.freeradbiomed.2012.10.554. 23127782

[ref50] Bettinger C. J. Materials advances for next-generation ingestible electronic medical devices. Trends Biotechnol. 2015, 33 (10), 575–585. 10.1016/j.tibtech.2015.07.008. 26403162

[ref51] Kalantar-Zadeh K.; Berean K. J.; Burgell R. E.; Muir J. G.; Gibson P. R. Intestinal gases: Influence on gut disorders and the role of dietary manipulations. Nat. Rev. Gastroenterol. Hepatol. 2019, 16 (12), 733–747. 10.1038/s41575-019-0193-z. 31520080

[ref52] Purohit H. J. Gut-bioreactor and human health in future. Indian J. Microbiol. 2018, 58 (1), 3–7. 10.1007/s12088-017-0697-6. 29434391 PMC5801186

[ref53] Ley R. E.; Peterson D. A.; Gordon J. I. Ecological and evolutionary forces shaping microbial diversity in the human intestine. Cell 2006, 124 (4), 837–848. 10.1016/j.cell.2006.02.017. 16497592

[ref54] Curvello R.; Raghuwanshi V. S.; Garnier G. Engineering nanocellulose hydrogels for biomedical applications. Adv. Colloid Interface Sci. 2019, 267, 47–61. 10.1016/j.cis.2019.03.002. 30884359

[ref55] Yang J.; Xu F.; Han C.-R. Metal ion mediated cellulose nanofibrils transient network in covalently cross-linked hydrogels: Mechanistic insight into morphology and dynamics. Biomacromolecules 2017, 18 (3), 1019–1028. 10.1021/acs.biomac.6b01915. 28192670

[ref56] Ferreira F. V.; Otoni C. G.; De France K. J.; Barud H. S.; Lona L. M.F.; Cranston E. D.; Rojas O. J. Porous nanocellulose gels and foams: Breakthrough status in the development of scaffolds for tissue engineering. Mater. Today 2020, 37, 126–141. 10.1016/j.mattod.2020.03.003.

[ref57] Du H.; Liu W.; Zhang M.; Si C.; Zhang X.; Li B. Cellulose nanocrystals and cellulose nanofibrils based hydrogels for biomedical applications. Carbohydr. Polym. 2019, 209, 130–144. 10.1016/j.carbpol.2019.01.020. 30732792

[ref58] Jayaramudu T.; Ko H.-U.; Kim H. C.; Kim J. W.; Muthoka R. M.; Kim J. Electroactive Hydrogels Made with Polyvinyl Alcohol/Cellulose Nanocrystals. Materials 2018, 11 (9), 1615 10.3390/ma11091615. PMC6163614 30181521

[ref59] Zubik K.; Singhsa P.; Wang Y.; Manuspiya H.; Narain R. Thermo-responsive poly (N-isopropylacrylamide)-cellulose nanocrystals hybrid hydrogels for wound dressing. Polymers 2017, 9 (4), 119 10.3390/polym9040119. PMC6432186 30970798

[ref60] Kabir S. M. F.; Sikdar P. P.; Haque B.; Bhuiyan M. A. R.; Ali A.; Islam M. N. Cellulose-based hydrogel materials: Chemistry, properties and their prospective applications. Progress in Biomaterials 2018, 7 (3), 153–174. 10.1007/s40204-018-0095-0. 30182344 PMC6173681

[ref61] Lewis L.; Hatzikiriakos S. G.; Hamad W. Y.; MacLachlan M. J. Freeze-Thaw Gelation of Cellulose Nanocrystals. ACS Macro Lett. 2019, 8 (5), 486–491. 10.1021/acsmacrolett.9b00140. 35619375

[ref62] Athukoralalage S. S.; Balu R.; Dutta N. K.; Roy Choudhury N. 3D Bioprinted Nanocellulose-Based Hydrogels for Tissue Engineering Applications: A Brief Review. Polymers 2019, 11 (5), 898 10.3390/polym11050898. PMC6572377 31108877

[ref63] Chau M.; Sriskandha S. E.; Pichugin D.; Thérien-Aubin H.; Nykypanchuk D.; Chauve G.; Méthot M.; Bouchard J.; Gang O.; Kumacheva E. Ion-Mediated Gelation of Aqueous Suspensions of Cellulose Nanocrystals. Biomacromolecules 2015, 16 (8), 2455–2462. 10.1021/acs.biomac.5b00701. 26102157

[ref64] Dorishetty P.; Balu R.; Athukoralalage S. S.; Greaves T. L.; Mata J.; De Campo L.; Saha N.; Zannettino A. C.; Dutta N. K.; Choudhury N. R. Tunable biomimetic hydrogels from silk fibroin and nanocellulose. ACS Sustainable Chem. Eng. 2020, 8 (6), 2375–2389. 10.1021/acssuschemeng.9b05317.

[ref65] Dorishetty P.; Balu R.; Sreekumar A.; de Campo L.; Mata J. P.; Choudhury N. R.; Dutta N. K. Robust and tunable hybrid hydrogels from photo-cross-linked soy protein isolate and regenerated silk fibroin. ACS Sustainable Chem. Eng. 2019, 7 (10), 9257–9271. 10.1021/acssuschemeng.9b00147.

[ref66] Varanasi S.; Henzel L.; Sharman S.; Batchelor W.; Garnier G. Producing nanofibres from carrots with a chemical-free process. Carbohydr. Polym. 2018, 184, 307–314. 10.1016/j.carbpol.2017.12.056. 29352924

[ref67] Lavoine N.; Bergström L. Nanocellulose-based foams and aerogels: Processing, properties, and applications. J. Mater. Chem. A 2017, 5 (31), 16105–16117. 10.1039/C7TA02807E.

[ref68] Mariano M.; Hantao L. W.; da Silva Bernardes J.; Strauss M. Microstructural characterization of nanocellulose foams prepared in the presence of cationic surfactants. Carbohydr. Polym. 2018, 195, 153–162. 10.1016/j.carbpol.2018.04.075. 29804963

[ref69] Levin D.; Saem S.; Osorio D. A.; Cerf A.; Cranston E. D.; Moran-Mirabal J. M. Green Templating of Ultraporous Cross-Linked Cellulose Nanocrystal Microparticles. Chem. Mater. 2018, 30 (21), 8040–8051. 10.1021/acs.chemmater.8b03858.

[ref70] Chau M.; De France K. J.; Kopera B.; Machado V. R.; Rosenfeldt S.; Reyes L.; Chan K. J. W.; Förster S.; Cranston E. D.; Hoare T.; Kumacheva E. Composite hydrogels with tunable anisotropic morphologies and mechanical properties. Chem. Mater. 2016, 28 (10), 3406–3415. 10.1021/acs.chemmater.6b00792.

[ref71] Berkelman T. 4 Generation of pH gradients. In Handbook of Isoelectric Focusing and Proteomics; Garfin D., Ahuja S., Eds.; Academic Press: Cambridge, MA, 2005; Separation Science and Technology, Vol. 7, pp 69–92, 10.1016/S0149-6395(05)80007-8.

[ref72] Bjellqvist B.; Ek K.; Giorgio Righetti P.; Gianazza E.; Görg A.; Westermeier R.; Postel W. Isoelectric focusing in immobilized pH gradients: Principle, methodology and some applications. J. Biochem. Biophys. Methods 1982, 6 (4), 317–339. 10.1016/0165-022X(82)90013-6. 7142660

[ref73] Figeys D. Proteomics: The basic overview. Industrial Proteomics: Applications for Biotechnology and Pharmaceuticals; Wiley: Hoboken, NJ, 2005; pp 1–62.

[ref74] Byrd B.; Tran H. Two-Dimensional Gel Electrophoresis with Immobilized pH Gradients. In Electrophoretic Separation of Proteins; Kurien B., Scofield R., Eds.; Humana Press: New York, 2019; Methods in Molecular Biology, Vol. 1855, pp 125–129, 10.1007/978-1-4939-8793-1_13. 30426414

[ref75] Nami Y.; Lornezhad G.; Kiani A.; Abdullah N.; Haghshenas B. Alginate-Persian Gum-Prebiotics microencapsulation impacts on the survival rate of Lactococcus lactis ABRIINW-N19 in orange juice. LWT 2020, 124, 109190 10.1016/j.lwt.2020.109190.

[ref76] Albadran H. A.; Monteagudo-Mera A.; Khutoryanskiy V. V.; Charalampopoulos D. Development of chitosan-coated agar-gelatin particles for probiotic delivery and targeted release in the gastrointestinal tract. Appl. Microbiol. Biotechnol. 2020, 104, 5749–5757. 10.1007/s00253-020-10632-w. 32377900 PMC7306021

[ref77] Lee H. A.; Bong Y.-J.; Kim H.; Jeong J.-K.; Kim H.-Y.; Lee K.-W.; Park K.-Y. Effect of nanometric Lactobacillus plantarum in kimchi on dextran sulfate sodium-induced colitis in mice. J. Med. Food 2015, 18 (10), 1073–1080. 10.1089/jmf.2015.3509. 26305853

[ref78] Yilmaz M. T.; Taylan O.; Karakas C. Y.; Dertli E. An alternative way to encapsulate probiotics within electrospun alginate nanofibers as monitored under simulated gastrointestinal conditions and in kefir. Carbohydr. Polym. 2020, 244, 116447 10.1016/j.carbpol.2020.116447. 32536387

[ref79] Rahmati F. Microencapsulation of Lactobacillus acidophilus and Lactobacillus plantarum in Eudragit S100 and alginate chitosan under gastrointestinal and normal conditions. Appl. Nanosci. 2020, 10 (2), 391–399. 10.1007/s13204-019-01174-3.

[ref80] Santos Monteiro S.; Albertina Silva Beserra Y.; Miguel Lisboa Oliveira H.; Pasquali M. A. d. B. Production of Probiotic Passion Fruit (Passiflora edulis Sims f. flavicarpa Deg.) Drink Using Lactobacillus reuteri and Microencapsulation via Spray Drying. Foods 2020, 9 (3), 335 10.3390/foods9030335. PMC7143088 32178366

[ref81] Fung W.-Y.; Yuen K.-H.; Liong M.-T. Agrowaste-Based Nanofibers as a Probiotic Encapsulant: Fabrication and Characterization. J. Agric. Food Chem. 2011, 59 (15), 8140–8147. 10.1021/jf2009342. 21711050

[ref82] Frakolaki G.; Giannou V.; Kekos D.; Tzia C. A review of the microencapsulation techniques for the incorporation of probiotic bacteria in functional foods. Crit. Rev. Food Sci. Nutr. 2021, 61, 1515–1536. 10.1080/10408398.2020.1761773. 32400195

[ref83] Rodrigues F.; Cedran M.; Bicas J.; Sato H. Encapsulated probiotic cells: Relevant techniques, natural sources as encapsulating materials and food applications-a narrative review. Food Res. Int. 2020, 137, 109682 10.1016/j.foodres.2020.109682. 33233258

[ref84] Sarao L. K.; Arora M. Probiotics, prebiotics, and microencapsulation: A review. Crit. Rev. Food Sci. Nutr. 2017, 57 (2), 344–371. 10.1080/10408398.2014.887055. 25848935

[ref85] Yao M.; Xie J.; Du H.; McClements D. J.; Xiao H.; Li L. Progress in microencapsulation of probiotics: A review. Compr. Rev. Food Sci. Food Saf. 2020, 19 (2), 857–874. 10.1111/1541-4337.12532. 33325164

[ref86] Fijałkowski K.; Peitler D.; Rakoczy R.; Żywicka A. Survival of probiotic lactic acid bacteria immobilized in different forms of bacterial cellulose in simulated gastric juices and bile salt solution. LWT—Food Sci. Technol. 2016, 68, 322–328. 10.1016/j.lwt.2015.12.038.

[ref87] Huq T.; Fraschini C.; Khan A.; Riedl B.; Bouchard J.; Lacroix M. Alginate based nanocomposite for microencapsulation of probiotic: Effect of cellulose nanocrystal (CNC) and lecithin. Carbohydr. Polym. 2017, 168, 61–69. 10.1016/j.carbpol.2017.03.032. 28457464

[ref88] Nguyen H.-T.; Truong D.-H.; Kouhoundé S.; Ly S.; Razafindralambo H.; Delvigne F. Biochemical engineering approaches for increasing viability and functionality of probiotic bacteria. Int. J. Mol. Sci. 2016, 17 (6), 867 10.3390/ijms17060867. PMC4926401 27271598

[ref89] Lacroix C.; Grattepanche F.; Doleyres Y.; Bergmaier D. Immobilised cell technologies for the dairy industry. In Applications of Cell Immobilisation Biotechnology; Nedović V., Willaert R., Eds.; Springer: Dordrecht, Netherlands, 2005; Focus on Biotechnology, Vol. 8B, pp 295–319, 10.1007/1-4020-3363-X_18.

[ref90] Żur J.; Wojcieszyńska D.; Guzik U. Metabolic responses of bacterial cells to immobilization. Molecules 2016, 21 (7), 958 10.3390/molecules21070958. PMC6273605 27455220

[ref91] Mitropoulou G.; Nedovic V.; Goyal A.; Kourkoutas Y. Immobilization technologies in probiotic food production. J. Nutr. Metab. 2013, 2013, 1–15. 10.1155/2013/716861. PMC3830840 24288597

